# Impact of Altered Intestinal Microbiota on Chronic Kidney Disease Progression

**DOI:** 10.3390/toxins10070300

**Published:** 2018-07-19

**Authors:** Esmeralda Castillo-Rodriguez, Raul Fernandez-Prado, Raquel Esteras, Maria Vanessa Perez-Gomez, Carolina Gracia-Iguacel, Beatriz Fernandez-Fernandez, Mehmet Kanbay, Alberto Tejedor, Alberto Lazaro, Marta Ruiz-Ortega, Emilio Gonzalez-Parra, Ana B. Sanz, Alberto Ortiz, Maria Dolores Sanchez-Niño

**Affiliations:** 1 Nephrology Department, IIS-Fundación Jiménez Díaz-Universidad Autónoma de Madrid, 28040 Madrid, Spain; ecastillor@quironsalud.es (E.C.-R.); raul.fernandezp@quironsalud.es (R.F.-P.); raquel.esteras@quironsalud.es (R.E.); mvanessa@fjd.es (M.V.P.-G.); CGraciaI@quironsalud.es (C.G.-I.); bfernandez@fjd.es (B.F.-F.); mruizo@quironsalud.es (M.R.-O.); egparra@quironsalud.es (E.G.-P.); asanz@fjd.es (A.B.S.); aortiz@fjd.es (A.O.); 2 Department of Internal Medicine, Koc University School of Medicine, Istanbul 34450, Turkey; drkanbay@yahoo.com; 3 Nefrología, IIS-Gregorio Marañón, Universidad Complutense de Madrid, 28007 Madrid, Spain; atejedor@senefro.org (A.T.); alazaro10@gmail.com (A.L.)

**Keywords:** chronic kidney disease, microbiota, choline, carnitine, tryptophan, tyrosine, trimethylamine N-Oxide, p-cresyl sulphate, indoxyl sulphate, gut-kidney axis

## Abstract

In chronic kidney disease (CKD), accumulation of uremic toxins is associated with an increased risk of CKD progression. Some uremic toxins result from nutrient processing by gut microbiota, yielding precursors of uremic toxins or uremic toxins themselves, such as trimethylamine N-Oxide (TMAO), p-cresyl sulphate, indoxyl sulphate and indole-3 acetic acid. Increased intake of some nutrients may modify the gut microbiota, increasing the number of bacteria that process them to yield uremic toxins. Circulating levels of nutrient-derived uremic toxins are associated to increased risk of CKD progression. This offers the opportunity for therapeutic intervention by either modifying the diet, modifying the microbiota, decreasing uremic toxin production by microbiota, increasing toxin excretion or targeting specific uremic toxins. We now review the link between nutrients, microbiota and uremic toxin with CKD progression. Specific focus will be placed on the generation specific uremic toxins with nephrotoxic potential, the decreased availability of bacteria-derived metabolites with nephroprotective potential, such as vitamin K and butyrate and the cellular and molecular mechanisms linking these toxins and protective factors to kidney diseases. This information provides a conceptual framework that allows the development of novel therapeutic approaches.

## 1. CKD and CKD Progression

Around 10% of the adult population has chronic kidney disease (CKD), which is diagnosed when the estimated glomerular filtration rate (eGFR) falls below 60 mL/min/1.73 m^2^ or the urinary albumin:creatinine ratio (UACR) rises above 30 mg/g or there is other evidence of kidney injury, for more than 3 months, even in the presence of normal GFR [1].The thresholds for eGFR and albuminuria are not arbitrary: they signal the point when kidney injury results in both a higher risk of death and in a higher risk of CKD progression to end-stage kidney disease (ESKD) [2,3]. The drivers of CKD progression and accelerated aging when albuminuria is present but eGFR is preserved are unclear, although recently, albuminuria was reported to decrease the expression of the anti-aging and nephroprotective protein of kidney origin Klotho [4]. However, accumulation of uremic toxins has been considered a key contributor to the consequences of CKD when eGFR is <60 mL/min/1.73 m^2^ [5]. Despite this, their relative contribution to CKD progression is less well characterized than the role of other factors, such as persistence of the kidney insult, compensatory hyperfiltration, albuminuria and inflammation [6,7]. We have now focused on the current understanding of how microbiota-derived molecules may contribute to or protect from CKD progression.

## 2. The Intestinal Microbiota in CKD

There is experimental and clinical evidence derived from both genomic and non-genomic studies suggesting an abnormal microbiota composition in CKD (reviewed in [8,9,10]). Among factors potentially impacting on microbiota composition, we find diet changes, prescribed drugs, accumulation of toxins that may alter the gut microenvironment and the impaired immune response which may disturb the host:microbiota interaction and lead to repetitive antibiotic courses. Indeed, there are reports of an increase in bacterial species prone to proteolytic fermentation, such as *Clostridium* and *Bacteroides* and/or a decrease in bacteria that may be protective or release potentially nephroprotective molecules (e.g., short chain fatty acids), such as *Lactobacillus*. Understanding the specific changes in the gut microbiota in uraemia, and above all the factors driving these changes, may help to design novel therapeutic interventions aimed at restoring a more physiological or nephroprotective gut microbiota. In this regard, if the driving factors persist, any attempt to modulate the microbiota by prescribing “microbiota supplements” are likely to fail. Novel therapeutic approaches have become available that may provide a way around traditional stumbling blocks (e.g., well-tolerated potassium-lowering agents may replace a vegetable-poor diet for hyperkalaemia) [11,12]. Additionally, understanding the specific microbiota-derived molecules involved in driving CKD or in protecting from CKD may help design specific therapeutic interventions aimed at decreasing or boosting the production and/or activity of such molecules, or modify the microbiota.

## 3. Potential Pathogenic Links between an Altered Intestinal Microbiota and CKD Progression

**Figure 1:**
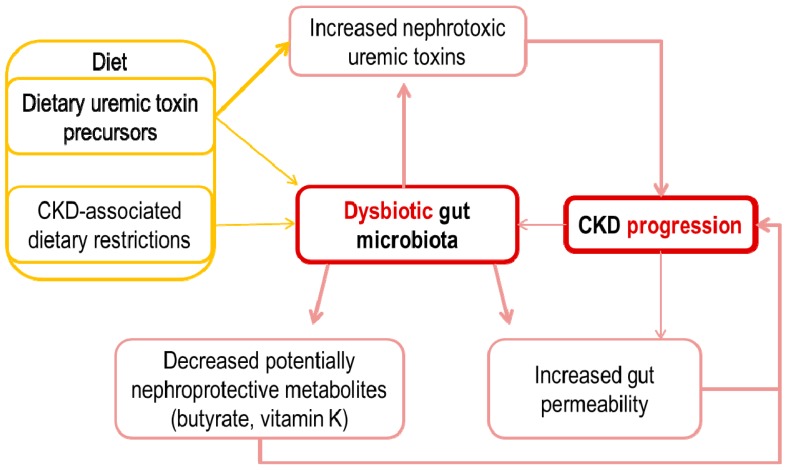
Bidirectional relationship between gut microbiota and chronic kidney disease (CKD) progression that may result in acceleration of CKD progression.

A number of pathogenic links between the intestinal microbiota, especially if this is altered and CKD progression have been described. These include an increased gut permeability, an overload of microbiota-generated potential nephrotoxins, such as p-cresyl sulphate (pCS), p-cresyl-glucuronide (pCG), indoxyl sulphate (IS), indole-3 acetic acid (IAA) and trimethylamine N-oxide (TMAO) [13] and decreased levels of certain microbiota-derived molecules that may have nephroprotective properties, such as butyrate and vitamin K (Table 1, Figure 1).

## 4. Increased Gut Permeability and Inflammation

CKD is associated to local and systemic inflammation and evidence of systemic inflammation has been associated with worse survival and kidney outcomes [38,39]. Drivers of inflammation include a decreased renal clearance of cytokines and inflammatory mediators as well as increased production of inflammatory mediators [40]. The gastrointestinal tract is a major source of chronic inflammation in CKD, both because of uremic toxins released by the microbiota, as discussed below and of an increased gut permeability that allows access of bacterial products to the circulation [41,42,43,44].

Circulating gut-derived bacterial DNA has been detected in patients with CKD and correlated with increased plasma C-reactive protein (CRP) and interleukin-6 (IL-6) [45,46,47]. Moreover, circulating levels of endotoxin, which is derived from the cell wall of Gram-negative bacteria, correlates with the severity of systemic inflammation in the absence of infection [48,49,50]. Although most studies have been performed in dialysis patients and circulating bacterial DNA is most frequent as CKD progresses, it was also found in non-dialysis CKD [50].

In cultured enterocytes, uremic plasma decreased transepithelial electrical resistance, denoting increased epithelial permeability [51]. This was accompanied by significant reductions in trans-cellular (i.e., claudin-1 and occluding) and intra-cellular (i.e., zonula occludens, ZO1) epithelial tight junction protein. Urea was identified as one of the potential drivers of these changes [52]. Additionally, oedema, hypervolemia or ischemia may disrupt the epithelial barrier. Finally, disruption of the physiological gut microbiota-enterocyte cross-talk as a result of an altered microbiota or defective innate immunity response because of uraemia, may also damage the gut epithelial barrier [53].

## 5. Metabolite Overload: Microbiota-Generated Nephrotoxins

Uremic toxins originating in the microbiota that have been linked in epidemiological studies and preclinical interventional studies to CKD progression include pCS and pCG, IS and IAA and TMAO.

### 5.1. pCS and pCG

p-cresol is synthesized by intestinal anaerobic bacteria from the dietary amino acids tyrosine and phenylalanine. Subsequent endogenous sulphate conjugation in the liver yields pCS and glucuronide conjugation in the enterocyte yields pCG, both normally excreted in urine through tubular secretion [54]. In CKD G1–G5 patients, total pCS and pCG accumulated to median levels of 50 μM (IQR, 21–104) and 0.22 μM (IQR, 0.08–0.60), respectively, reaching in individual G5 patients values up to 500 and 8 µM, respectively [5,14]. That is, pCS reaches levels around 200-fold higher than pCG [15]. With advancing CKD, the pCS to pCG ratio decreases and both higher total p-cresol (pCS+pCG) and a lower pCS to pCG ratio are independently associated to mortality [15]. However, most pCS and pCG is protein-bound and median free levels are 30- and 2-fold lower than total levels, respectively.

pCS has been associated with and may contribute to overall mortality, cardiovascular disease and progression of CKD [16,17]. While vascular effects may eventually negatively impact CKD progression, pCS also has direct effects on kidney cells, as recently reviewed [18]. Specifically, in kidney proximal tubular cells, pCS is cytotoxic and activates oxidative stress and proinflammatory and profibrotic responses, while decreasing nephroprotective factors such as Klotho [18,19,55]. Long-term (7 days) exposure to pCS induced apoptosis in cultured human proximal tubule cells in a concentration-dependent manner [19]. In addition, short-term (3 h) exposure to pCS promoted the expression of the TWEAK receptor Fn14, cooperated with TWEAK in promoting cell death and increased inflammatory gene expression. As expected, albumin alone was cytotoxic to proximal tubular cells [20]. Additionally, albumin also increased the inflammatory response to pCS concentrations found in the circulation of non-dialysis CKD patients. In contrast, no biological actions of pCG were observed on human proximal tubule cell inflammatory responses, either alone or in combination with pCS [19]. Probenecid decreased pCS-induced toxicity in proximal tubular cells, suggesting the requirement for active pCS transport into cells for the cytotoxic effects [55].

Both pCS and pCG decreased the function or the activity of proximal cell membrane transporters. pCS decreased the activity of multidrug resistance protein 4 (MRP4/ABCC4) and breast cancer resistance protein (BCRP/ABCG2), while pCG only decreased MRP4 [56]. Additionally, both pCS and pCG promoted evidence of epithelial-to-mesenchymal transition (EMT) and pCS promoted tubular damage and kidney fibrosis in vivo [55,56,57]. In fact, pCS activated the renal-angiotensin system in vivo and kidney fibrosis was decreased by losartan [57].

### 5.2. IS and IAA

IS is a small protein-bound molecule [58]. Bacterial tryptophanases in the colon converted dietary tryptophan to indole which is absorbed into the systemic circulation. Indole is further metabolized by the liver to form IS, which is then cleared by the kidneys through tubular secretion mediated by organic anion transporter OAT1 and OAT3. Total IS accumulates in CKD to a highest individual reported total value of 1100 µM and highest reported mean value of 211 µM, versus 2 µM in controls [18]. By contrast, free IS is undetectable in controls and maximum values in CKD are around 20 µM [18].

IS may contribute to vascular and renal disease progression. Although vascular effects may eventually negatively impact CKD progression, IS also has direct effects on kidney cells, that have been more intensively explored than the nephrotoxic effects of other toxins, as recently reviewed [18]. In cultured tubular cells, four key effects were observed: increased oxidative stress, increased inflammatory (e.g., chemokine secretion) and profibrotic (e.g., EMT, intrarenal renal-angiotensin system activation, increased TGF-β1 levels and activity) responses and decreased expression of nephroprotective factors (e.g., Klotho) [18,21,22]. Additionally, IS administration to subtotally nephrectomised rats accelerated fibrosis and CKD progression, as did its precursor indole [23,24,25,26]. Immunohistochemistry located IS to proximal and distal tubules, which express OAT1 (proximal tubules) and/or OAT3 (proximal and distal tubules), suggesting that IS accumulation in uraemia may facilitate tubular cell overload of the toxin [23]. In this regard, IS decreased the viability of cultured proximal tubular cells in an OAT1- and OAT3-dependent manner and this was inhibited by the OAT inhibitor probenecid [23]. Probenecid is routinely used in the clinic to reduce cidofovir nephrotoxicity by preventing entry into proximal tubular cells, an effect reproducible in cultured cells [59]. Interestingly, cilastatin also decrease tubular cell uptake of IS, although the effect on toxicity was not explored. Cilastatin protects cultured tubular cells and the kidneys in vivo from diverse nephrotoxic agents [60]. Cilastatin is also in clinical use to extend the half-life on certain β-lactam antibiotics (such as imipenem) by decreasing tubular uptake. IS also injures podocytes, perturbing the actin cytoskeleton, decreasing the expression of podocyte-specific genes and modulating the inflammatory response in culture and in vivo and causing albuminuria [61].

IAA is a protein-bound small molecule also derived from tryptophan metabolism and excreted in urine by tubular secretion through the OAT1 transporter [62]. Serum total IAA levels increase in patients with CKD as eGFR decreases; the mean uremic concentration is about 5 µM and the highest concentration is 50 µM [5]. In subtotally nephrectomised rats, the oral intake of 125 mg/kg/day IAA resulted in a higher excretion of *N*-acetyl-β-glucosiminadase (NAG), a marker of tubular injury, a lower GFR and higher glomerular sclerosis index, indicating that an excess of IAA on top of previously injured kidneys may accelerate CKD progression [28]. However, there is less information on actions of IAA on cultured renal cells. In porcine proximal tubular LLC-PK1, IAA at a concentration of 250 µM reduced viability mainly through induction of apoptosis [27]. However, this concentration is not found in vivo.

Both IS and IAA activate the transcription factor aryl hydrocarbon receptor (AhR), which regulates the cell response to environmental xenobiotics and promotes vascular inflammation and oxidative stress [63]. At concentrations found in the circulation of CKD patients, both molecules increased tissue factor expression in endothelial cells, vascular smooth muscle cells and peripheral blood mononuclear cells by increasing its stability through AhR signalling [64,65,66]. Tissue factor is an initiator of coagulation and AhR antagonists behave as antithrombotic agents in the CKD context [66]. Additional effects have been described in endothelial cells, including oxidative stress and dysfunction. In this regard, in cultured human endothelial cells, IAA activated an inflammatory non-genomic AhR/p38MAPK/NF-κB pathway that induced endothelial inflammation and oxidative stress [63]. Whether activation of AhR also account for adverse effects on tubular cells and podocytes is still unknown. At least some actions of IS on proximal tubular cells, such as downregulation of the Mas receptor, are indeed mediated by AhR [67]. Additional nuclear translocation of AhR was observed in podocytes, although whether this mediated IS-induced responses was not explored [61].

### 5.3. TMAO

TMAO is generated from trimethylamine (TMA)-containing nutrients present in seafood, or from TMA released by the gut microbiota. Dietary TMA precursors, such as choline, phosphatidylcholine (lecithin), or l-carnitine are metabolized by gut microbiota to generate TMA. Then, TMA is absorbed and metabolized in the liver by Flavin-containing monooxygenases (FMO1 and FMO3) to yield TMAO [68,69,70]. TMAO is eventually cleared mainly by the kidneys, excreted in urine in part through tubular cell secretion involving uptake by organic cation transporters (OCTs) OCT1 and OCT2 as well as transporters of the ATP-binding cassette (ABC) family, including ABCG2 (BCRP) and ABCB1 (MDR1) [71,72]. Multidrug and toxin extrusion protein 1 (MATE1) contributes to translocation of TMAO across the luminal membrane of proximal tubular cells [73]. Indeed, genetic ABCG2 variants modulate TMAO exposure in humans [72]. TMAO may also be metabolically retroconverted to TMA by certain gut bacteria, such as Enterobacteriaceae and this influences the microbiota composition [74]. Although not specifically studied, this process may be especially relevant in CKD, given the high availability of TMAO. Additionally, TMAO may also be excreted in sweat and exhaled air [13]. Haemodialysis efficiently removes TMAO [75]. The physiological role of TMAO in humans is unclear [76]. In some marine animals, it behaves as an osmolyte that maintains cell volume and counteracts the destabilizing effect of high urea concentration and hydrostatic pressure on proteins structures that inhibit ligand binding [77].

Circulating TMAO levels are elevated in subjects with impaired renal function. Plasma TMAO was higher among CKD G3–G5 patients (eGFR < 60 mL/min/1.73 m^2^) than in non-CKD patients (7.9; IQR 5.2–12.4 versus 3.4; IQR 2.3–5.3 µM, *p* < 0.001) [29]. In another study, median TMAO levels in CKD G4 was higher than in G3b patients (25.15; IQR 16.64–38.56 versus 14.32; IQR 9.14–22.69 µM) [30]. TMAO decrease following renal transplantation [78]. TMAO levels correlated with evidence of systemic inflammation and was an independent predictor of mortality and CVD in CKD patients [29,30,78,79]. TMAO may also contribute to the pathogenesis and progression of CKD [29,80]. In a metabolomic study on plasma from 1434 participants (Framingham Heart Study) with normal renal function at baseline, elevated choline and TMAO levels predicted the development of CKD [80].

The biological plausibility of cause-and-effect relationship between high TMAO levels and adverse kidney outcomes in human CKD has been addressed in experimental animals [29]. In mice fed a diet supplemented with choline (1.0%) or TMAO (0.12%) for 6 weeks, circulating TMAO levels increased as compared to a normal diet (0.08% choline) to levels (40, 100 and <5 µM, respectively) found in human CKD [29]. Mice fed the high choline or TMAO diets developed progressive renal tubulointerstitial fibrosis and dysfunction and TMAO levels correlated with kidney tubulointerstitial fibrosis and collagen deposition, increased phosphorylation of SMAD3, which signals the profibrotic activity of TGF-β1 and increased kidney injury molecule-1 (KIM-1) [29]. In an independent study, feeding mice a high fat diet increased TMAO levels as well as kidney tubulointerstitial fibrosis and collagen deposition, SMAD3 phosphorylation, KIM-1, plasma cystatin C and evidence of inflammation, such as NOX-4 and TNF-α. The inhibitor of TMA formation 3,3-Dimethyl-1-butanol (DMB) prevented these effects [81]. Thus, animal data are suggestive of a deleterious effect of TMAO on CKD. However, in the absence of cell culture studies, it is unclear whether this is a direct toxic effect of TMAO on renal cells or whether it is mediated by systemic effects of TMAO or the adaptive response to the diets used in the mice studies.

## 6. The Missing Ones: Microbiota-Generated Nephroprotective Factors

### 6.1. Vitamin K 

Vitamin K refers to a group of fat-soluble vitamins required for the carboxylation of glutamate residues to form gamma-carboxyglutamate (Gla) residues, which are required for the function of, among others, proteins involved in coagulation and in the control of tissue calcification. Concomitant with carboxylation, reduced vitamin K (KH_2_) is oxidized to vitamin K epoxide (KO). KO must be recycled back to KH_2_ by the enzymes vitamin K epoxide reductase and vitamin K reductase in a pathway known as the vitamin K cycle [82]. An anti-inflammatory action has been proposed that is independent of its role as an enzyme co-factor [83]. There are two natural vitamers: vitamin K1 (phylloquinone), made by plants and found in green leafy vegetables and K2 (menaquinone) synthesized by bacteria in the gut and in fermented foods. Menaquinones have side chains of varying length and are generally denoted as MK-n, where n stands for the number of isoprene residues in the side chain, which differ in bioavailability, half-life and biodistribution [84]. The adequate intake level for vitamin K is estimated at 70 to 120 μg/day. However, it is thought that vegetable vitamin K1 is poorly bioavailable and contributes <10% of absorbed vitamin K, thus increasing the dependence on gut microbiota generated menaquinone. There is recent interest in subclinical vitamin K deficits, characterized by reduced carboxylation of Gla proteins that are mild enough not to prolong the prothrombin time.

In CKD, several defects related to vitamin K have been reported. In CKD G3–G5 patients not on dialysis with a mean age of 61 years, mean vitamin K1 levels were 2.1 ± 2.4 nM, which is in the range found in non-CKD patients (e.g., 1.54 ± 2.0 nM was reported for general population individuals with a mean age of 59 years) [85,86]. Despite this apparently normal vitamin K1 level, there was evidence of subclinical vitamin K deficiency, as defined by a percentage of uncarboxylated osteocalcin (ucOC) >20%, which is a more sensitive marker of vitamin K status. In CKD not on dialysis, ucOC was 27 ± 22% versus 16 ± 16% in healthy people [85,86]. In addition to potential gut microbiota derangements, that have been poorly studied for vitamin K, CKD patients appear to be prone to vitamin K deficiency for at least two reasons: a reduced dietary intake (diets low in potassium contain fewer leafy green vegetables; rich in K1 and diets low in phosphate contain fewer dairy products; rich in K2) and a reduced expression and activity of vitamin K 2,3-epoxide reductase (VKOR), the target of coumarins and other vitamin K-related enzymes [87].

Carboxylated matrix Gla protein (MGP) is a potent inhibitor of arterial calcification and needs vitamin K for synthesis [88]. Assessing levels of inactive dephospho-uncarboxylatedMGP (dp-ucMGP) provides insights into potential vitamin K deficiency [89]. In patients with diabetes, renal dysfunction, or macrovascular disease, dp-ucMGP behaves as a circulating biomarker associated with cardiovascular risk, more severe illness, or higher mortality [88]. Median plasma dp-ucMGP levels in CKD stages 3, 4 and 5 are 586 (452–888), 870 (594–1591) and 1050 (518–1298) pM, respectively [90]. In rat kidney failure, activity of the rate-limiting enzyme in the vitamin K cycle—gamma-glutamyl carboxylase (GGCX)—was reduced, resulting in increased serum ucMGP levels [91]. Vitamin K2 supplementation, better than K1, resulted in significantly lower calcium content in kidneys and aorta, decreasing medial elastocalcinosis [91,92]. In this regard, oral anticoagulants inhibiting the C1 subunit of the vitamin K epoxidereductase enzyme complex are major contributors to the severe clinical condition calciphylaxis [93]. Uraemia was also associated with a decrease in kidney vitamin K1 to menaquinone K-4 (MK-4) bioconversion enzyme, UBIAD1. Supplementation of vitamin K1 or the MK-4 form of vitamin K2 at pharmacological doses restored the abnormal vitamin K cycle activity and slowed the progression of vascular calcification [87]. In this regard, there is increasing concern about the potential negative effects of vitamin K targeting oral anticoagulants, such as warfarin and coumarin, on vascular calcification, given their frequent use in CKD patients with valvular calcification that required valve replacement or is complicated by atrial fibrillation and the increased sensitivity of these patients to vascular calcification [94].

More recently, a negative impact of warfarin on renal function has been described. Initial reports described haematuria-induced acute kidney injury (AKI) related to over coagulation [95]. Indeed, oral anticoagulation is a frequent driver of IgA nephropathy-associated AKI driven by macroscopic haematuria episodes [96]. Then, the association of transient over coagulation with increases in creatinine levels was observed, mainly in but not only on CKD patients [97]. More recently, more rapid CKD progression has been described in individuals anticoagulated with warfarin than in users of direct anticoagulants [98]. Thus, while macroscopic haematuria–associated AKI has been observed with any oral anticoagulant [99,100], drugs targeting vitamin K appear to increase the risk of CKD progression. Whether this is related to a nephroprotective action of vitamin K, the more frequent over coagulation episodes with warfarin or nephroprotective effects of direct anticoagulants related to factor Xa inhibition remains to be clarified. Surprisingly, Framingham Offspring study participants in the highest vitamin K1 quartile (≥1.78 nM) had an increased risk of CKD, whereas no association was observed with continuous vitamin K1 levels [92]. These data are weak as different results were obtained with different analyses and require validation. Additionally, assessing vitamin K2 may have been more informative, given that it comprises a higher portion of absorbed vitamin K.

The potential role of vitamin K disturbances in CKD progression and the consequences for dietary counselling, modulation of the microbiota, use of vitamin K supplements and choice of oral anticoagulants merits further studies.

### 6.2. Butyrate

Butyrate is a short-chain fatty acid produced by anaerobic human intestinal microbiota and is the preferred energy source for colon epithelial cells [101]. Butyrate is formed from two molecules of acetyl CoA which are converted, via the intermediates β-hydroxybutyryl CoA and crotonyl CoA, to butyryl CoA and finally to butyrate. The main bacteria butyrate-releasing are *Faecalibacterium prausnitzii, Roseburia intestinalis* and some *Clostridium*-related bacteria [33].

Butyrate concentrations in venous blood are very low. There are no estimates in CKD but in the general population it has been estimated at 2–4 µM or 35–65 µM by different authors [31,32]. A circadian rhythm has been described, with lower values after breakfast and transient increases after lunch and dinner, which have been ascribed to dietary fibre fermentation. In the intestinal lumen, butyrate concentrations are much higher, around 5–20 mM but this is not a good guide to production rates because of the very high colonic mucosa absorption [33]. Lower numbers of butyrate-producing bacteria were observed in human ESKD [102,103,104] but the concentration or uptake of butyrate in this population has not been measured yet.

Butyrate is a general inhibitor of histone deacetylases (HDAC) and protects against high-fat diet-induced metabolic changes [105], has anti-inflammatory properties [106], extends lifespan in experimental progeria mice [107] and protects from features of aging such as sarcopenia, which are prominent in CKD [108]. HDACs are key epigenetic regulators that have been linked to kidney injury and downregulation of Klotho expression [109,110]. Interestingly, the butyrate intermediary crotonyl CoA may be a source of crotonate, another epigenetic regulator which protects from kidney injury [6,111]. Additionally, like other short chain fatty acids, butyrate activates the GPR43 receptor [112].

In vivo, butyrate protects from kidney injury in animal models. The daily administration of 50 to 200 mg/kg sodium butyrate intraperitoneal for 8 days attenuated gentamicin-induced nephrotoxicity in rats in a dose-dependent manner [36]. This was ascribed to the inhibitory activity of butyrate on HDAC, decreased oxidative stress and increased expression of the chaperon prohibitin [36]. However, none of the experiments performed demonstrated that either the HDAC inhibitory activity or prohibitin expression were required for the nephroprotective effect. In experimental contrast-induced nephropathy in rats, administration of 500 mg/kg sodium butyrate intravenously decreased the severity of renal failure, assessed by serum creatinine and of systemic inflammation, assessed by serum IL-6 levels and NF-κB activation in the kidney [37].

The protective effect of butyrate has been confirmed in culture mouse glomerular mesangial cells, where it dampened the release of monocyte chemoattractant protein-1 (MCP-1), IL-1β and intercellular adhesion molecule-1 (ICAM-1) and decreased oxidative stress in response to lipopolysaccharide and high glucose [34]. However, the effective concentration, at 5 mM, is two to three orders of magnitude higher than butyrate concentrations in the circulation and unlikely to be reached by modulating butyrate production in the gut. In this regard, butyrate concentrations that are relevant for enterocytes may not be relevant for kidney cells. In human cultured renal cortical epithelial cells, 500 µM butyrate reduced MCP-1 mRNA in response to TNF-α by 18% and by 29% at 1000 µM [35]. Again, these concentrations are one or two orders of magnitude higher than in the circulation.

## 7. Nephroprotection Interventional Studies Targeting the Intestinal Microbiota or Microbiota-Derived Toxins

**Figure 2:**
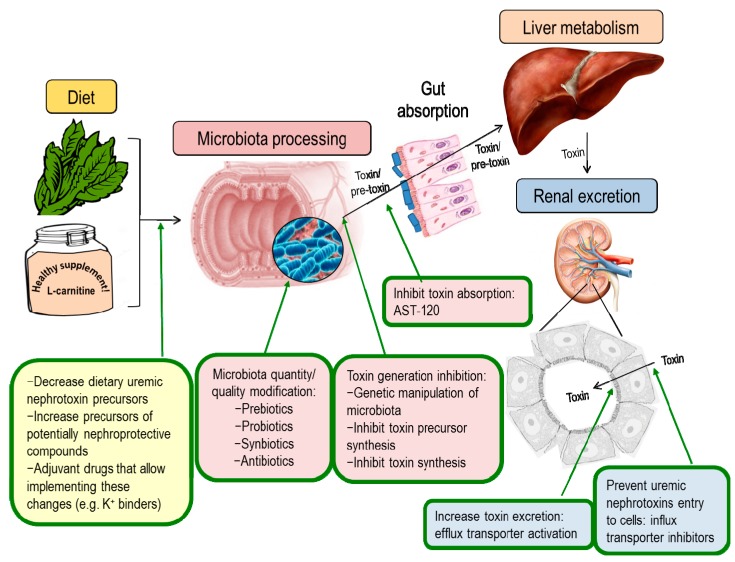
Potential therapeutic approaches on the gut microbiota-CKD progression axis. Only one of these approaches is used routinely in the clinic in some countries (AST-120). The rest are theoretical or have been tested only in preclinical cell culture or animal models.

Several strategies have been tested in animal models to target microbiota-derived toxins and their nephrotoxicity, including reduction of uremic toxin synthesis, increasing the excretion by modulating the expression or activity of renal transporters, preventing gut absorption by using oral adsorbents and modulation of the microbiota composition (Figure 2). Of these, only the latter two have reached the clinical stage.

### 7.1. Modulation of Uremic Toxins Synthesis

A dietary restriction of uremic toxin precursors is a theoretical approach to limit uremic toxin synthesis. Despite the fact that superimposing additional restrictions on patients potentially suffering from anorexia and at high malnutrition risk seems impractical, some precursor of uremic toxins with nephrotoxin potential are sold as over-the-counter food supplements or prescription drugs and both patients and physicians should be aware of the potential dangers [113] (reviewed in [13], Table 2).

Genetic manipulation of bacteria may be used to reduce their ability to synthesize uremic toxin precursors. The overall safety of this strategy should be carefully explored, including the potential consequences for bacteria pathogenicity. In this regard, successful application of such a therapy would require that bacteria are able to transfer the genetic modification horizontally or that it creates a survival advantage over other non-genetically modified microbiota competitors. In this sense, BT1492 was identified as the more abundant tryptophanase in human gut microbiota and one mutation in this gene reduces indol synthesis and IS levels in mice [114]. Moreover, IS levels in mice were reduced by antibiotics that favour the growth of BT1492 negative bacteria [114].

Phytochemical polyphenols, such as quercetin and resveratrol, reduce synthesis of IS through inhibition of sulfotransferase (SULT), an enzyme implicates in IS synthesis in liver [115]. These drugs have proved nephroprotective in a variety of animal models but present pleiotropic actions and the contribution of IS synthesis inhibition to any nephroprotective effect remains unproven, even if reduced IS levels are observed, as is the case for cisplatin-induced AKI, since a better renal function would already be expected to decrease IS levels [115]. In addition, both drugs also protected from ischemia/reperfusion renal injury but in this case resveratrol did not reduce renal IS, questioning whether IS reduction contributes to nephroprotection [116].

Among a library of different drugs, meclofenamate, a non-steroidal anti-inflammatory drug, (NSAID) decreased IS synthesis. Meclofenamate reduced IS levels in serum, kidney and liver and protected from ischemia/reperfusion renal injury [117]. Given the adverse renal and cardiovascular safety profile of NSAIDs, their use to decrease uremic toxin synthesis seems unwarranted. However, the molecule may be modified to increase its efficiency in decreasing IS production while decreasing its NSAID profile. Even in this case, the safety of long-term suppression of IS production by the liver, itself a pathway for toxin metabolism and clearance, should be demonstrated.

### 7.2. Modulation of Renal Transporters

Proximal tubular cell transporters facilitate the entry of protein-bound uremic toxins into proximal tubular cells and their subsequent tubular secretion. Specifically, organic anion transporters of the OAT (SLC21) and OATP (SLC22/SLCO) families from the basolateral membrane and ATP-binding cassette (ABC) transporters such as MDR1, MRP2 and MRP4 in the luminal membrane have been implicated in the excretion of clinically relevant uremic toxins and may decrease when CKD progresses [118]. While in cultured cells, inhibition of these transporters with drugs such as probenecid decreased the cytotoxicity of uremic and non-uremic toxins [55,59], an increased activity of these transporters in vivo may increase kidney toxin clearance. The final result in vivo over kidney injury is not easy to predict. On one hand decreasing circulating levels of toxins through increased tubular secretion may decrease any adverse effects of these toxins over non-kidney cells. However, increasing transtubular traffic of the toxins may be detrimental for tubular cells. As an in vivo proof-of-concept, in transgenic rats, SLCO4C1 (SLC21A20) overexpression decreased kidney inflammation and hypertension following subtotal nephrectomy [118]. However, kidney function was not improved and tissue injury was not reported and was likely unchanged. Nephroprotection was associated to decreased plasma levels of uremic toxins such as guanidino succinate, asymmetric dimethylarginine and trans-aconitate but not of other better characterized nephrotoxic uremic toxins, such as IS [119]. Interestingly, SLCO4C1 expression is regulated by AhR: IS downregulates and statins increase SLCO4C1 expression through interaction with AhR [119,120]. Thus, while SLCO4C1 overexpression was not associated with clinically significant preservation of renal function in rat CKD, it provides insights into how to manipulate the activity of proximal tubular transporters to accelerate the clearance of uremic toxins that may accelerate CKD progression. In this regard, meclofenamate increased the expression of the organic anion transporters OAT1 and OAT3, favouring IS excretion in rats [117].

### 7.3. Adsorbents 

AST-120 is an oral carbonic adsorbent which is used to decrease the absorption of uremic toxins, such as indols and retard CKD in Japan [121]. In experimental CKD, it reduces tissue accumulation of IS and pCS in diverse tissues, including the kidney and protected the kidneys in several animal models of AKI and CKD [122], However, two large multinational trials (EPPIC-1 and EPPIC-2), failed to show benefit on CKD progression [123]. However, a post-hoc, hypothesis-generating analysis observed that in the subgroup of high risk patients (urinary creatinine ratio ≥1.0 and haematuria) who were additionally treated with renin-angiotensin system blockade, AST-120 may have provided additional benefit in retarding CKD progression [124]. The large daily number of pills may negatively impact compliance and may be one of the underlying cause for the failure of clinical trials outside Japan.

### 7.4. Modulation of Microbiota Composition

Another approach to modulate uremic toxin production is the therapeutic manipulation of the composition of the gut microbiota. Several ongoing clinical trials are exploring the impact of changing the diet or using prebiotics (non-digestible food ingredients), probiotics (living microorganisms) or synbiotics (a mixture of both compounds) on microbiota composition [13]. In experimental animals, antibiotics decreased the production of uremic toxins [125]. It could be argued that they are frequently used in CKD patients. The potential adverse effects of antibiotic use for this purpose, both over the individual patient and on public health may outweigh the potential benefits. However, chronic non-absorbable antibiotics are clinically indicated for other conditions associated with microbiota-generated toxins, such as hepatic encephalopathy and clinical trials in CKD that provide information into risk:benefit balance may be worthy.

Hyperkalaemia is favoured by both progressive CKD and nephroprotective drugs such as renin-angiotensin system blockers. Besides limiting the use of these medications, it may trigger the prescription of low potassium diets. This usually entails limiting foods that promote a healthy microbiota such as fruits and vegetables. While the impact of low potassium diets as prescribed for CKD patients on the microbiota, uremic toxins levels and CKD progression has not been studied in detail, the recent availability of a new generation of drugs for hyperkalaemia, such as patiromer and zirconium cyclosilicate [11,12], offers the opportunity of designing clinical trials that address these issues.

The use of a low protein diet supplemented with essential amino acid and ketoacid supplements may offer some advantages in CKD patients but the impact on the gut microbiota and uremic toxin production is poorly characterized [126]. An ongoing trial (NCT02302287) is testing the impact of Mediterranean diet, ketoacid-supplemented low protein diet or a combination on the composition of the gut microbiota in CKD patients. Resistant starch, a class fermentable fibre, may be used as a prebiotic and has beneficial effects on obesity-related diabetes [127]. An ongoing clinical trial (NCT02706808) is exploring the impact of resistant starch on microbiota composition and uremic toxin levels in CKD patients.

Advanced glycation end-products (AGEs) may also promote kidney disease [128]. This is specially an issue in diabetic patients, in whom high glucose levels lead to increased levels of glucose-degradation products and in peritoneal dialysis patients, due to continuous glucose and glucose-degradation product absorption from the peritoneum [129]. Additionally, a number of processed foods are a source of dietary AGEs [130]. In this regard, a recent trial of dietary restriction of AGEs in peritoneal dialysis patients demonstrated a decrease in serum AGEs as well as a change in bacterial microbiota [129]. At baseline, peritoneal dialysis patients exhibited a lower relative abundance of *Bacteroides* and *Alistipes* genus and a higher abundance of *Prevotella* genus when compared to the published data of healthy population. Dietary AGE restriction decreased *Prevotella copri* and *Bifidobacterium animalis* relative abundance and increased *Alistipes indistinctus*, *Clostridium citroniae, Clostridium hathewayi* and *Ruminococcus gauvreauii* relative abundance [129].

Some studies have addressed the impact of oligosaccharide prebiotics on uremic toxin levels, with mixed success but none has addressed the impact on CKD progression. In haemodialysis patients, oligofructose inulin decreased p-cresol (pCS+pCG)) but not IS [13,131], while a synbiotic (*Lactobacillus casei*, *Bifidobacterium breve* and galacto-oligosaccharide) decreased pCS [13,132]. In CKD patients not on dialysis, arabinoxylan oligosaccharides did not modify the urinary excretion of pCS, pCG, IS or TMAO [131]. It is likely that the impact of any given prebiotic or synbiotic will depend on the baseline characteristics of the individual, including baseline dietary intake of oligosaccharides and uremic toxin precursors and microbiota composition. Additional clinical trials are addressing the impact of multispecies probiotic mixes on gut permeability and microbiota composition in CKD patients [133].

## 8. The Way Forward

Understanding the impact of an altered intestinal microbiota on CKD progression, be it through promotion of inflammation related to an increased gut permeability of through the generation of diet-derived nephrotoxic uremic toxins or their precursors or through decreased availability of nephroprotective molecules, has the potential to greatly impact the management of CKD and pave the way for novel therapeutic approaches to slow progression of CKD [3,8,41] (Figure 2). We should also remember that even in dialysis patients, slowing the progressive loss of residual renal function may have a substantial clinical impact, because residual renal function is inversely related to mortality and a benefit has been observed not only in peritoneal dialysis but also in haemodialysis [134,135]. The range of potential intervention spans from diet modification to population-level education efforts regarding the potential impact of food supplements to reducing uremic toxin generation rate and intestinal absorption, modifying the microbiome or design of interventions to limit toxin actions, such as targeting the AhR [9,136,137]. A detailed discussion of modulation of uremic toxins is provided by Koppe et al. in this issue [138]. In this regard, some drugs successfully tested preclinically in culture or in vivo, are already in clinical use, although sometimes for a different indication. For example, in cultured proximal tubular cells IS toxicity required toxin uptake by tubular transporters sensitive to probenecid and cilastatin and these two drugs are in clinical use and indeed, one of the clinical uses of probenecid is to prevent nephrotoxicity [59]. Additionally, a comprehensive understanding of the different factors involved, from diet to individual microbiota composition to a permissive genetic background to either generate higher toxin levels or facilitating toxin access to target renal cells or sensitizing to their toxicity, may facilitate a personalized approach, taking into account the multiple variables that impact on uremic toxins levels and, likely, the individual sensitivity to them. This may also help put into perspective the often-higher concentrations used in preclinical studied as compared to circulating levels in CKD patients. A systems biology approach, including microbiome and metabolomics analysis exploring individual characteristics regarding the microbiota and the uremic toxin profile should be explored in targeted populations in this regard. This information may yield a relatively low number of key data that may help individualize and monitor novel therapeutic approaches in a wider population.

## Figures and Tables

**Table 1:** Key uremic toxins of bacterial origin that may promote CKD progression and bacterial products with potential nephroprotective effects. Some of these toxins are protein-bound, mainly albumin-bound. Despite tubular cells not being physiologically exposed to high albumin concentrations, they actively uptake some of these compounds and intracellular concentrations may be higher than in other cell types.

Compound	Total Plasma Concentration in CKD G4/G5 Non-Dialysis (µM)	Lowest Concentration Active on Cultured Renal Cells (µM)	Effects on Cultured Renal Cells	Effects on Kidneys In Vivo	References
pCS	Median 50 Maximum 500	100	Decreased viability, increased oxidative stress, increased inflammatory and profibrotic responses, decreased expression of nephroprotective factors and viability, decreased function of (MRP4/ABCC4 and BCRP/ABCG2) in tubular cells.	Progression of CKD, kidney fibrosis Promote epithelial-to-mesenchymal transition Activate the renal-angiotensin system	[5,14,15,16,17,18,19,20]
pCG	Median 0.22 Maximum 8	25	No actions observed on human proximal tubule cell inflammatory response Decreased the function of proximal cell membrane transporters (MRP4)	Kidney fibrosis Promotes epithelial-to-mesenchymal transition	[5,14,15]
IS	Median 211 Maximum 1100	1000	Decreased viability, increased oxidative stress, increased inflammatory and profibrotic responses, decreased expression of nephroprotective factors and viability, nuclear AhR translocation in tubular cells. Podocyte injury	Accelerated fibrosis and CKD progression Podocyte injury	[18,21,22,23,24,25,26]
IAA	Median 5 Maximum 50	250	Reduced viability through induction of apoptosis in tubular cells	Accelerated CKD progression	[27,28]
TMAO	Median 25 Upper quartile > 38	ND	No data	Kidney tubulointerstitial fibrosis	[29,30]
Butyrate	Not data in CKD patients 2–65 in blood 5000–20,000 in gut lumen in non-CKD	500–1000	Reduced inflammation	Protects from gentamicin and contrast nephrotoxicity	[31,32,33,34,35,36,37]

AhR: aryl hydrocarbon receptor; CKD: chronic kidney disease; IAA: indole-3 acetic acid, IS: indoxyl sulphate; pCS: p-cresyl sulphate; pCG: p-cresyl-glucuronide; TMAO: trimethylamine N-oxide.

**Table 2:** Over-the-counter food supplements or prescription drugs with the potential to generate uremic toxins with nephrotoxic effects. Information obtained from reference [13]. Putative benefit or indications refers to the reasons sellers use to peddle the supplement. It does not imply that we endorse these benefits or indications.

Supplement	Putative Benefits or Indications	Resulting Uremic Toxins with Nephrotoxicity Potential
l-tyrosine (para-tyrosine, 4-hydroxyphenylalanine)	Enhanced physical performance, enhanced cognitive performance,	pCS, pCG
Tryptophan	Antidepressant, anxiolytic, sleep aid	IS, IAA
Choline/phosphatidylcholine/lecithin	Liver health, memory, Alzheimer disease, enhanced physical performance, pregnancy	TMAO
l-carnitine	Enhanced physical performance, haemodialysis	TMAO

pCS: p-cresyl sulphate, pCG: p-cresyl-glucuronide, IS: indoxyl sulphate, IAA: indole-3 acetic acid, TMAO: trimethylamine N-oxide.
